# The time dependent influence of curvature and topography of biomaterials in the behavior of corneal endothelial cells

**DOI:** 10.3389/fbioe.2024.1454675

**Published:** 2024-09-25

**Authors:** Begoña M. Bosch, Luis M. Delgado, Raquel Rodríguez-González, Roman A. Perez

**Affiliations:** ^1^ Bioengineering Institute of Technology, Universitat Internacional de Catalunya, Barcelona, Spain; ^2^ Bioengineering Department, Universitat Internacional de Catalunya, Barcelona, Spain

**Keywords:** cornea, corneal endothelia cells, biomaterials, tissue engineeering, corneal endothelium

## Abstract

Among the different layers of the cornea, the corneal endothelium, which is composed of corneal endothelial cells (CEC), plays a key role in the maintenance of cornea transparency. Based on the donor shortages and the limitations associated with transplantation, in this work we have developed collagen hydrogels with different patterned structures on the surface to provide topographies in ranges similar to the natural environment that CEC sense. This aimed at stimulating cells to maintain a typical CEC phenotype and provide alternatives to corneal transplantation. In this sense, we have elaborated curved collagen hydrogels (concave and convex) with three different topographies (50, 200 and 300 µm grooves), with the help of 3D printed mold and replicating the mold with the collagen hydrogel, onto which CEC were cultured in order to analyze its behavior. Flat hydrogels were used as controls. Cell morphology, cell circularity and gene expression of ATP1A1 and ZO-1 genes were analyzed after 3 and 6 days. Results showed an effect of the curvature and the topography compared to flat collagen hydrogels, showing higher expression of ZO-1 and ATP1A1 in curved non-patterned hydrogels at day 3. The patterned hydrogels did not have such a significant effect on gene expression compared to flat hydrogels, showing stronger effect of the curvature compared to the topography. The effect was opposite at day 6, showing higher gene expression at days 6 on the patterned hydrogels, especially for the ZO-1 gene. The gene expression results were in accordance with the cell morphology observed at the different time points, showing circularities closer to hexagon like morphology at shorter time points. Overall, this platform can serve as a system to culture cell under proper environment to further be able to transplant a CEC monolayer or to allow transplantation of thin collagen layers with cultured CEC.

## 1 Introduction

Diseases affecting the cornea are the fifth leading cause of blindness and it affects more than 10 million people in the world today (Mathews et al., 2018). Nowadays, corneal transplant is the gold standard treatment for these diseases. Corneal transplant, named as keratoplasty, can be performed replacing the full cornea, known as penetrating keratoplasty (PK), or only the damaged corneal layers, known as partial thickness transplants (Boynton and Woodward, 2015). Endothelial keratoplasties, which is performed upon corneal endothelium failure, is the most performed keratoplasty and represents more than 60% of all corneal transplants. The corneal endothelium (CE) plays a key role in the maintenance of cornea transparency through its barriers and the Na^+^/K^+^-ATPase pump functions (Labelle, 2016). The CE consists of a monolayer of polygonal cells, known as corneal endothelial cells (CEC), that are in a non-proliferative state (Joyce et al., 1996). In consequence, CEC loss or dysfunction can lead to loss of vision.

There are different diseases affecting the CE such as Fuchs Endothelial Corneal Dystrophy, Posterior Polymorphous Corneal Dystrophy or Congenital Hereditary Endothelial Dystrophy, among others. Regarding the diverse CE diseases, Fuchs Dystrophy is the most common one (Mathews et al., 2022; Jeang et al., 2021; Sarnicola et al., 2019) and represented more than 39% of all keratoplasties performed in the United States in 2022. However, surgical intervention is still a difficult option, and this process faces many challenges, such as a severe worldwide shortage of donors and graft rejection by the patient (Navaratnam et al., 2015). Hence, to address this issue, it is necessary to find alternative solutions, such as the development of an *in vitro* tissue construct for corneal endothelium (CE) regeneration.

In order to find strategies to regenerate or repopulate the CE, it is essential to mimic the extracellular matrix (ECM) that the cells sense. The CE is intimately bonded to the Descemet’s membrane, an ECM structure mainly composed of fibronectin, laminin, and collagen. The unique topography, elasticity, and surface charge of this ECM suggests direct implications on corneal endothelial cells (CEC) behavior. Hence, modifying the physical properties of substrates that may ultimately resemble the Descemet’s membrane is an interesting approach to control CEC behavior.

Previous works revealed the effect of *in vitro* microenvironments for corneal endothelium tissue engineering alternating the topography and surface properties of substrates. For instance, it was shown that patterned substrates in the form of concentric circles, wells and pillars enhanced human corneal endothelial cell (HCEC) proliferation, morphology and function (Koo et al., 2014; Muhammad et al., 2015a; Gutermuth et al., 2019). The enhanced results were based on higher proliferation rates, a more polygonal typical shape and higher protein and gene expression levels of the two main HCEC markers in patterned surfaces (Koo et al., 2014; Muhammad et al., 2015a; Gutermuth et al., 2019). Another study developed PDMS substrates with rose petal topography, presenting a functionalization either with hyaluronic acid or collagen IV, culturing bovine CEC on them for 7 days. Results showed a higher cell proliferation and higher expression of CEC-like marker Na^+^/K^+^-ATPase when cells were cultured with collagen four substrates and the patterned topography (Öztürk-Öncel et al., 2021). However, these previously presented works are based on the lithography technique, requiring specific materials, which are generally non-implantable and very specific set-ups together with a time-consuming and high-cost preparation technique (Gutermuth et al., 2019; Qin et al., 2010; Levinson, 2005). Despite the topography has revealed a positive effect on CEC behavior, this technology is generally based on a 2D approach and scaling the nanotopographies into a 3D system remains a challenge (Qin et al., 2010; Levinson, 2005).

Considering that corneal tissues have the native curvature of eyes, we hypothesized that culturing cells in a curved pattern could resemble the natural conditions in which CEC are found and hence contribute to a better cell behavior. Actually, it has been previously observed that different cellular types have been cultured on curved substrates and that these curvatures were able to significantly affect its phenotypes. Yoshida et al. demonstrated that HCEC could be cultured on a porcine-derived scaffold with spherical curvature, similar to that of the cornea (Yoshida et al., 2014). Results showed a regular distribution of the cells although phenotypic analyses were not performed.

Hence, we aim to combine, for the first time, the production of curved substrates with specific topographies using native corneal tissues mimicking materials. Initially, we studied the feasibility of generating curved and micropatterned structures using a conventional 3D printer for corneal endothelium tissue engineering. We hypothesized that the generation of a substrate that mimicked native ECM with curved morphologies and with specified microtopographies could modify cell phenotype which could have a beneficial effect on CEC behavior.

## 2 Materials and methods

### 2.1 Patterned hydrogel formation

#### 2.1.1 Mold design and fabrication

The anatomical eye model was extracted from a previous study (Garner, 1982) and the different concave, convex and flat molds were designed. The final dimensions were 11 mm of corneal diameter and a value of sagittal height of 2 mm (Garner, 1982). The resulting molds were exported as an STL file and, subsequently, the g-code for 3D printing was generated using the software Simplify3D v4.1 (Simplify3D).

Patterned molds were fabricated with poly-lactic acid (PLA) using a FDM 3D printer (BCN3D Technologies) with a 0.4 mm nozzle. Concave, flat and convex molds were printed using a concentric circle pattern. Due to the intrinsic properties of the layer-by-layer printing of the molds, a multistep wave was introduced over the curvature of the molds and the waveforms were controlled by adjusting the 3D printing layer heights (LH) (50, 200 and 300 μm). These different LH were provided to combine the curvature of the substrate with the substrate topography at different levels. As control, an unpatterned surface was used. In order to fabricate the unpatterned surfaced molds, the 50 μm LH molds were treated with acetone for 3 min and its surface was smoothed with the help of a microbrush. PLA molds were sterilized with 70% ethanol and UV irradiation for at least 30 min. Subsequently, molds were rinsed twice with 1X phosphate buffered saline (PBS) (Sigma-Aldrich).

#### 2.1.2 Collagen extraction

The platforms were fabricated using collagen type I, which were shaped into the proper morphology with the help of the designed PLA molds. Tendons from 16-month bovine were collected from a local abattoir. Type I collagen isolation protocol was performed as previously reported (Delgado et al., 2017a). Shortly, tendons were separated manually from its surrounding fascia, muscle or blood, and washed with 1X PBS solution. Tendons were kept at 4°C during the whole extraction process. The tendon tissue was crushed with a desktop blender and dissolved in 1 M acetic acid with continuous mild stirring up to 72 h. Then, pepsin was added at a ratio of 40 U per milligram of tendon and the solution was incubated for 72 h under agitation. Subsequently, insoluble tendon was filtered and discarded, while soluble collagen solution was kept stirring overnight. Collagen was then purified by repeated salt precipitation using 0.9 M sodium chloride (NaCl) (Sigma-Aldrich). Precipitated collagen was collected and incubated in 1 M acetic acid (Sigma-Aldrich) under gentle agitation for up to 72 h. Finally, collagen solution was dialyzed against 1 mM acetic acid using membranes with molecular weight cut-off of 12,000 (Sigma-Aldrich). Final collagen solution was placed in a sterile recipient and stored at 4°C. Sodium dodecyl sulphate polyacrylamide gel electrophoresis (SDS-PAGE) was performed in order to confirm collagen purity, as previously described (Delgado et al., 2017b).

#### 2.1.3 Patterned hydrogel

Firstly, the final collagen solution was combined with 10X PBS, which is required to increase the ionic salt solution and trigger collagen fibrillization. Then, the pH of the collagen solution was adjusted to pH = 7.2–7.4, initially with 25 µL of 5 M NaOH per ml of collagen to drastically increase the pH followed by the addition of tens of microliters of 1 M (10–100 µL per ml) NaOH to more accurately adjust the pH. In order to control the pH evolution and finalize the titration at the established pH, phenol red indicator was incorporated. Genipin, a low toxicity cross-linker (Delgado et al., 2017a), was added for the crosslinking of the hydrogel to achieve higher stability. The collagen was then centrifuged to remove bubbles, followed by the addition of 450 μL collagen solution onto the different designed molds placed in 48-well plates, incubating them at 37°C for 45 min for collagen gelation. The genipin solution was then added on the collagen hydrogel at a volume ratio of genipin:collagen of 1:200. Then, plates were sealed with parafilm and kept at room temperature overnight. The LH and distance between peaks were measured in order to analyze the platforms used for human corneal endothelial cells (HCEC) culture. Optical microscope images were taken (Olympus CKX41, Nikon) and characterization of curved patterned samples was performed using ImageJ. Finally, hydrogels were washed with sterile PBS and cell culture medium before cell seeding.

### 2.2 Cell culture

Cell culture was performed in two different stages. Initially, in order to observe the possible interaction between the patterned substrates and cells, fibroblast cells were placed on the designed platforms as preliminary experiments. This is based on the capacity of fibroblasts to easily proliferate and explore the substrates. Once the optimum conditions were found, CEC were used with the optimum conditions, as it is known that CEC are more sensitive and less proliferative.

#### 2.2.1 Fibroblasts culture: understanding the effect of topography size on cell morphology

Fibroblasts were cultured in fibroblast medium which contained DMEM with 4.5 g/L glucose supplemented with 10% FBS, 1% GlutaMax and 1% Penicillin-Streptomycin. The medium was changed every 2–3 days. When the cells reached 80% confluence, they were detached using TrypLE™ Express Enzyme and re-plated on the hydrogel. Cells were cultured at a seeding density of 2.5 × 10^4^ cells/cm^2^. Three LH of patterned flat hydrogels were used: 300, 200 and 50 μm. After 24 h, fibroblasts were fixed and cell morphology and density were analyzed by immunofluorescence assay.

#### 2.2.2 Immunofluorescence assay

Cell morphology was analyzed by immunofluorescence assay. Briefly, cells were washed with 1X PBS and fixed with 4% paraformaldehyde (Sigma-Aldrich) for 20 min at room temperature. Then, cells were permeabilized with 0.1% Triton X-100 for 10 min at room temperature. Cells were washed and stained with 100 nM of the conjugated antibody Acti-stain 488 phalloidin (Biogen, Cat. CY-PHDG1-A) for 30 min in the dark at room temperature. Subsequently, cells were washed and incubated with nuclear DAPI for 5 min. Finally, cells were rinsed with PBS and observed using an inverted fluorescence microscope (Leica Microsystems, GMi8).

#### 2.2.3 Corneal endothelial cell culture

Human Corneal Endothelial Primary cells were purchased from Celprogen and cultured following manufacturer’s recommendations. The primary cells present in this study were obtained from Celprogen. Shortly, frozen vial was centrifuged at 100 g for 7 min and resuspended in CEC commercial medium (Celprogen). All cells were plated in a T25 commercial culture flasks and incubated at 37°C and 5% CO_2_. Celprogen’s Human Corneal Endothelial Primary Cell Culture Complete Growth Medium was used for the experiments and was changed every 24–48 h. When cell confluence reached 95%, cells were detached using EDTA and centrifuged. Then, cells were re-plated at a cell density of 1 × 10^4^ cells/cm^2^, unless otherwise specified, or used for future experiments.

#### 2.2.4 Corneal endothelial cells on hydrogel: understating the effect of curvature and topography

Based on our preliminary results on fibroblast culture, cells were cultured on different unpatterned and patterned hydrogels at a seeding density of 2 × 10^5^ cells/cm^2^ which resembled native cell density. Cell morphology, cell size and total RNA were analyzed at days 3 and 6 of culture. Cell circularity and cell size was determined to evaluate the effect of the curvature and topography during 6 days of culture. Cell circularity was calculated according to the following formula.

$$Circularity=\frac{4\pi \times Area}{{Perimeter}^{2}}$$

Where a circularity value of 1 indicates a perfect circle, while a value of 0 corresponds to an elongated shape. Therefore, due to its characteristic polygonal-hexagonal shape, Ang et al. (2016) evaluated that *in vivo* CEC morphology has a circularity value close to 0.87–0.88. At least 100 cells per condition were analyzed.

#### 2.2.5 Quantitative real time–polymerase chain reaction (qPCR)

In order to understand the main key markers of CEC differentiation, several genes were analyzed at 3 and 6 days. Gene expression was analyzed by quantitative real time–polymerase chain reaction (qPCR). Shortly, total RNA was isolated using NucleoSpin RNA kit (Macherey-Nagel) including DNAse treatment following the manufacturer’s instructions. One µg of RNA with a ratio of intensities at the wavelengths of 260/280 nm between 1.8-2 was then reversed transcribed into cDNA using Transcriptor First Strand cDNA Synthesis Kit (Roche) according to the manufacturer’s recommendations. CEC specific primers (Table 1) and FastStart Universal SYBR Green Master (Roche) were used to amplify the desired cDNA. Finally, the amplifications were performed in a CFX96 Real-Time PCR Detection System (Bio-Rad) for quantitative Real-Time PCR.

**TABLE 1:** List of primers used for cDNA amplification in quantitative RT-PCR.

Gene	Primer	Sequence (5'-3′)	Accession Number
ATP1A1	Forward	TGT​GAT​TCT​GGC​TGA​GAA​CG	NM_001160234
	Reverse	TGC​TCA​TAG​GTC​CAC​TGC​TG	
ZO-1	Forward	AGT​TTG​GCA​GCA​AGA​GAT​GG	NM_001355015
	Reverse	GCT​GTC​AGA​AAG​ATC​AGG​GA	
b-ACTIN	Forward	AGA​GCT​ACG​AGC​TGC​CTG​AC	NM_001101
	Reverse	AGC​ACT​GTG​TTG​GCG​TAC​AG	

### 2.3 Statistical analysis

One way analysis of variance (ANOVA) followed was used after confirming normal distribution from each sample population (Shapiro-Wilk’s test for normality test) and equality of variances (Levene’s tests). Non-parametric statistics were used when either or both of the above assumptions were violated.

For molds characterization, cell circularity and cell area assay, one-way analysis of variance (ANOVA) was performed to analyze differences between groups followed by multicomparison tests, using Tukey’s test. A *p*-value of less than 0.05 was accepted as indicating statistical significance. The data are presented as mean ± SEM.

Regarding gene expression, Mann-Whitney *U*-test was carried out to reveal significant differences. Differences were considered statistically significant with *p*-value of less than 0.05. The data are presented as mean ± standard deviation.

GraphPad Prism version 6 (GraphPad Software Inc.) was used to graph the data and statistical analyses were performed using Statistical Package for the Social Sciences (SPSS) 21 (IBM).

## 3 Results

### 3.1 Patterned hydrogel characterization

**FIGURE 1:**
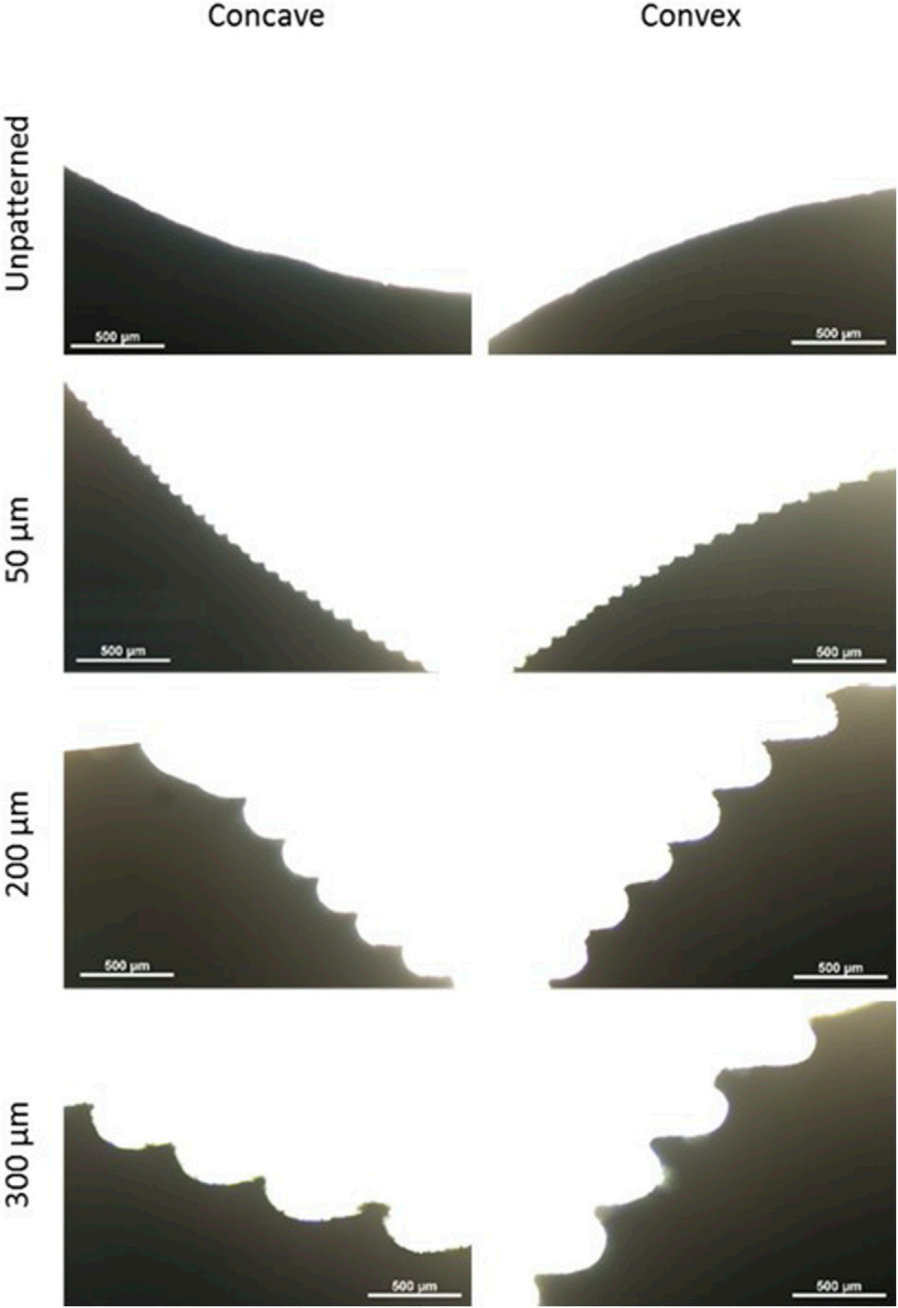
Cross-sections of the different samples. Concave and convex molds with printing resolutions of 300, 200 and 50 μm and unpatterned molds. Scale bars: 500 μm.

Initially, molds were designed and printed using different printing resolutions on a conventional 3D printer. Then, hydrogels were placed on the curved molds to obtain the desired patterns. In order to characterize the topography of the hydrogels, the cross-sections of the different samples were analyzed. As can be seen in Figure 1, unpatterned samples presented a smooth surface. On patterned models, microwells were formed between the peaks. The width of the different peaks increased as the LH was increased. When using the lower LH molds, the microwells significantly reduced their size compared to the higher LH molds.

**FIGURE 2:**
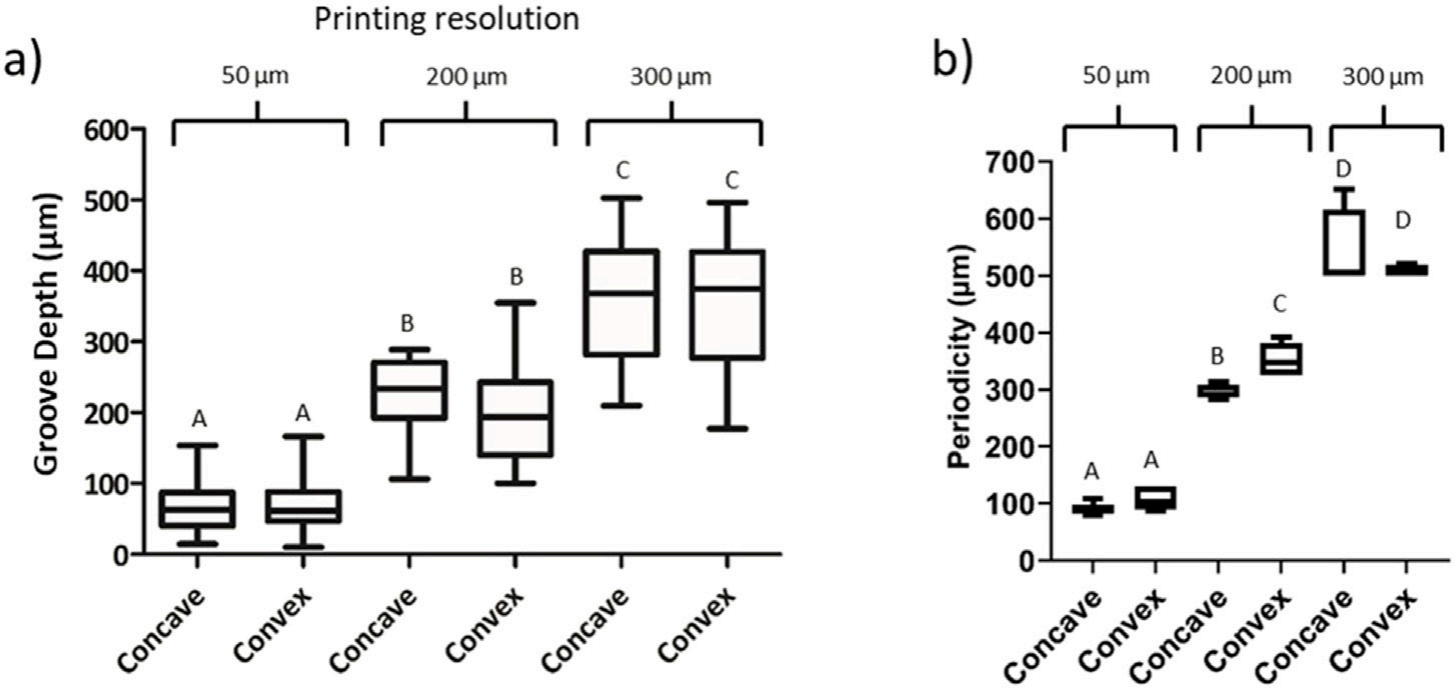
Image analysis of the topography created with the replica molding technique. **(A)** Groove depth measurement based on the printing resolution. **(B)** Periodicity of the grooves. Concave and convex molds were analyzed with printing resolutions of 50, 200 and 300 μm. Letters represent statistical significance. Columns presenting different letters represent significant differences (*p* < 0.05).

Furthermore, the distance between peaks and the LH was analyzed. As shown in Figure 2, the distance between peaks and the LH increased following the expected values. Moreover, the values between concave and convex molds were similar, although the values were more heterogeneous as the printing resolution increased.

### 3.2 Fibroblast culture on patterned hydrogel formation

#### 3.2.1 Effect of topography size

**FIGURE 3:**
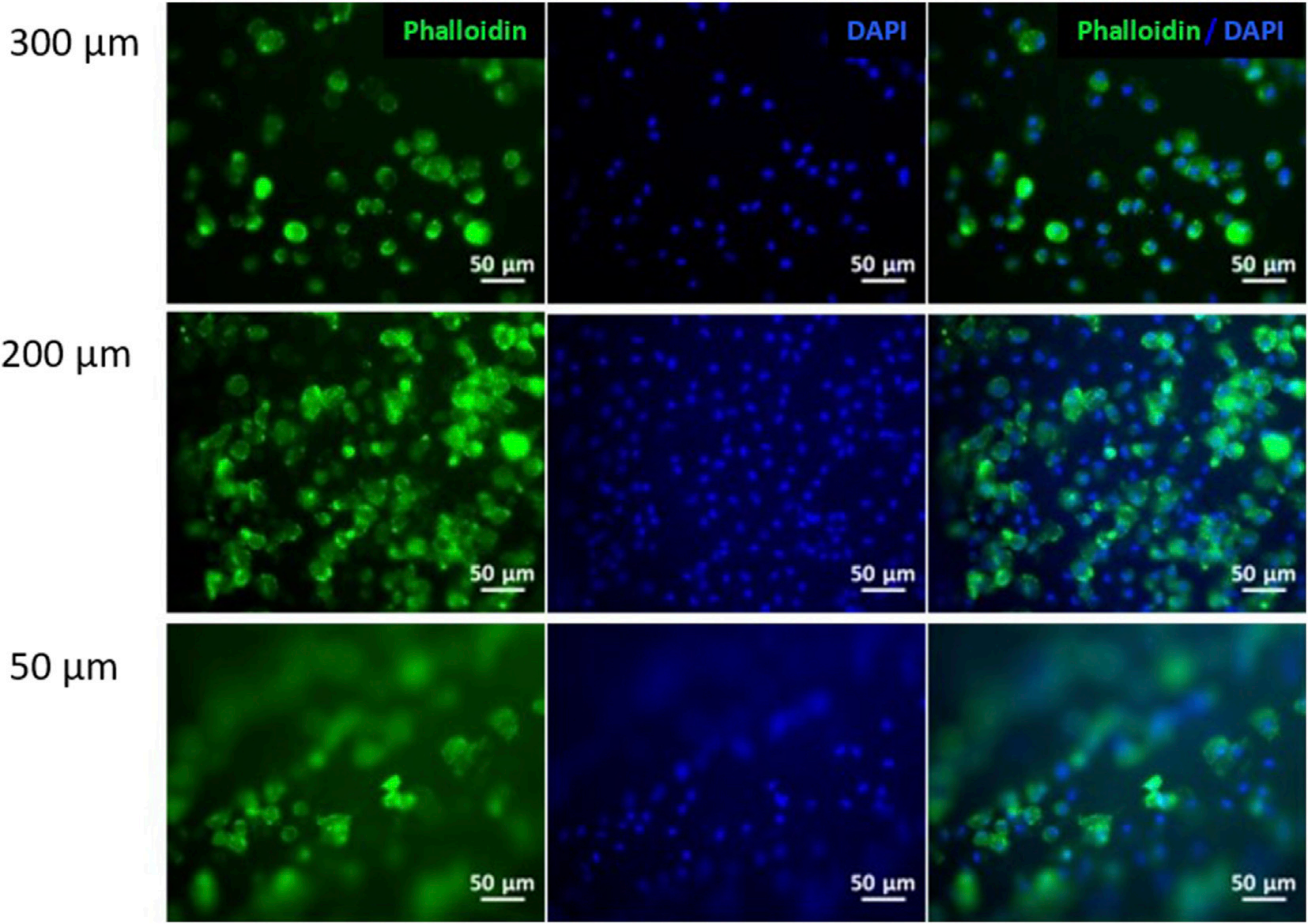
Fibroblasts cell culture on collagen hydrogels. Immunofluorescence analysis of phalloidin (green) and nuclear DAPI (blue) on patterned hydrogels at different layer heights: 300, 200 and 50 μm. Scale bars: 50 μm.

Initially, fibroblasts were cultured on the collagen flat substrates with the different LH in order to understand the effect of the size of topography on cell behavior. Figure 3 shows cells cultured on top of the hydrogels with high LH (300 μm), showing lower cell number as well as presenting a random cell distribution. Interestingly, the 300 μm LH presented the lowest cell number compared to the 200 and 50 μm LH. While the 200 and 50 μm LH presented similar cell number, the general trend showed a certain degree of alignment for the LH (50 µm) compared to the other heights. For this reason, the patterned hydrogel with 50 µm of LH was used for subsequent experiments.

### 3.3 CEC culture on patterned cross-linked hydrogels

**FIGURE 4:**
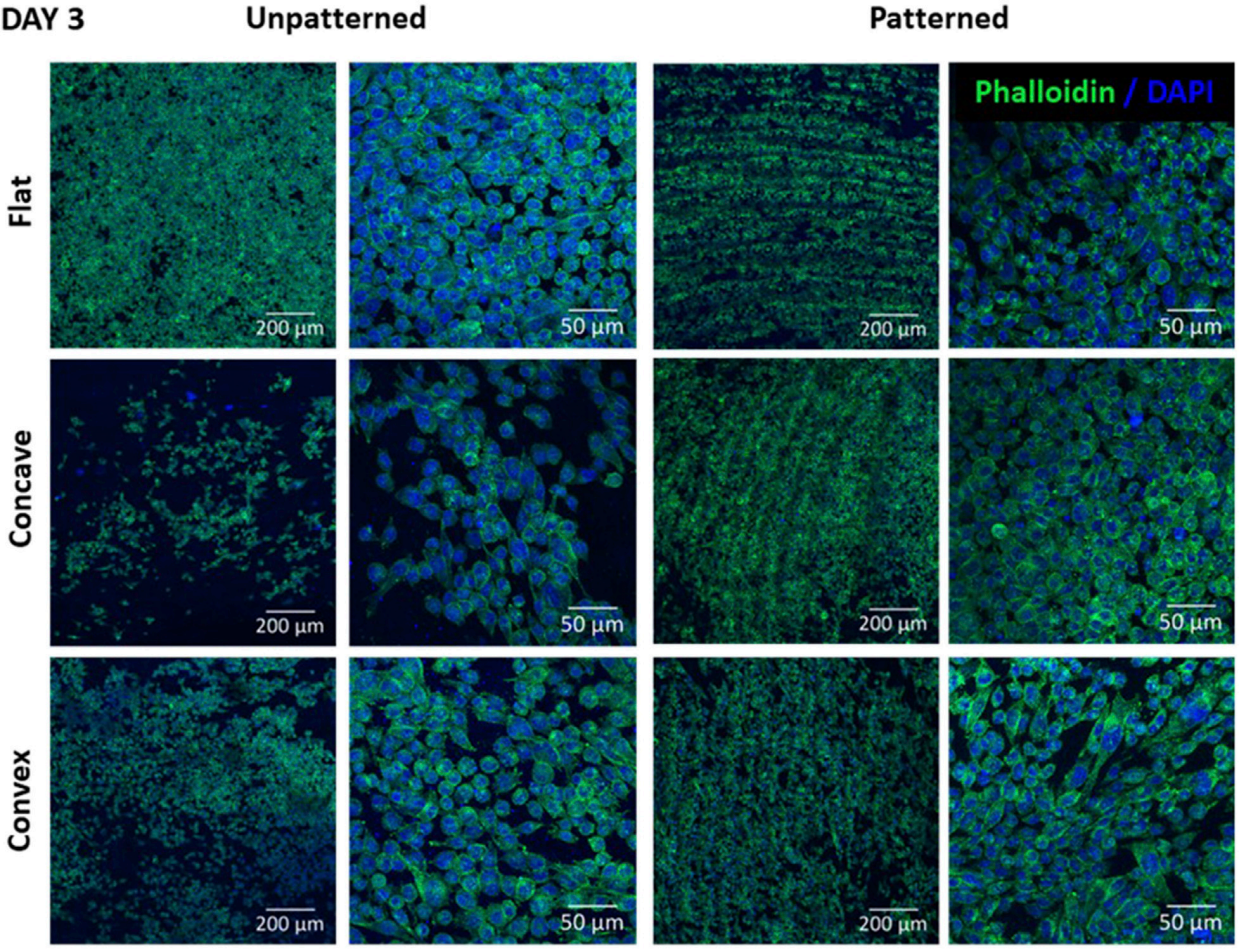
CEC cultured on collagen unpatterned and patterned hydrogels for 3 days. Cell morphology on flat, concave and convex surfaces, on both unpatterned and patterned hydrogels, analyzed by immunofluorescence analysis of phalloidin (green) and nuclear DAPI (blue). Scale bars: 200 μm (left) and 50 μm (right).

Figure 4 shows the morphology of CEC cultured on the different substrates after 3 days of culture. In the unpatterned substrates, the flat surface clearly showed a higher number of cells with a rather circular morphology. The convex hydrogel apparently presented slightly higher cell number compared to the concave hydrogel. Cell morphology presented similar morphology as in the flat surface. Nevertheless, this trend was not followed for the patterned surfaces, showing similar number of cells in all substrates. Furthermore, the flat surface presented rounder cells compared to the convex and concave surfaces, which had, apparently more polygonal cells in the concave surface and rather elongated morphology in the convex surface. Moreover, cells presented preferent alignment in the grooves, as observed in the low magnification images, although this cell alignment was not present at higher magnification.

**FIGURE 5:**
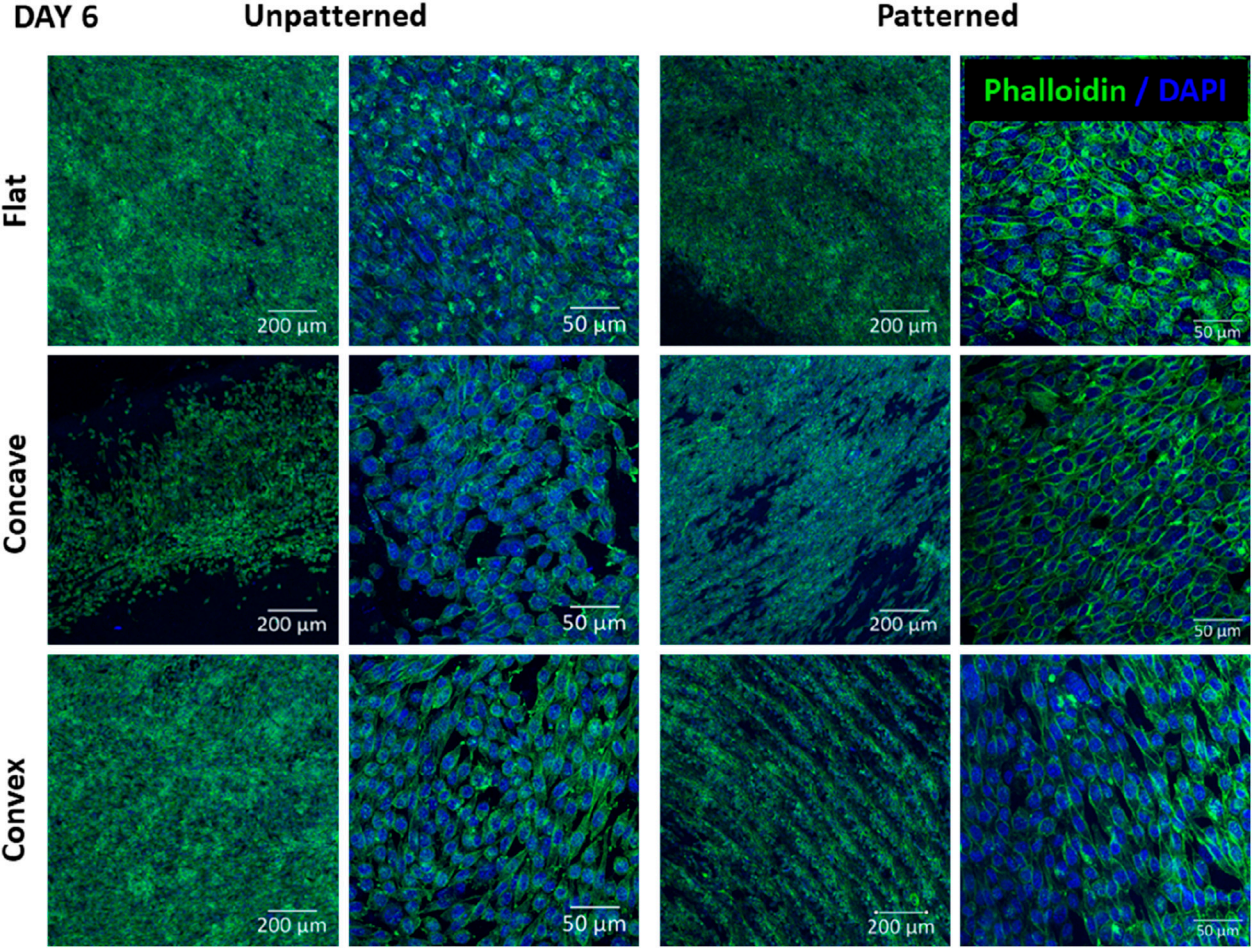
CEC cultured on collagen unpatterned and patterned hydrogels for 6 days. Cell morphology on flat, concave and convex surfaces, on both unpatterned and patterned hydrogels, analyzed by immunofluorescence analysis of phalloidin (green) and nuclear DAPI (blue). Scale bars: 200 μm (left) and 50 μm (right).

Figure 5 shows the cell morphology of CEC culture on the different substrates after 6 days of culture. Regarding the unpatterned substrates, the number of cells increased in all cases, although the differences among the different substrates were similar to those found at 3 days. Despite the flat surfaces still presented higher cell number, the convex substrate seemed to have higher cell number and confluence compared to the concave. Cell morphology was similar in the different cases, presenting rather elongated cells both in the flat and convex substrates and rather round cells in the concave substrates. Regarding the patterned substrates, cells tended to align on the substrate both in the convex and concave substrate but did no align in the flat surface. At higher magnification, the cell morphology showed some tendency to have polyhedric cells in all cases, having a more apparent rounded morphology for the cells in the flat and concave surfaces compared to the convex surface. Interestingly, both the convex and concave surfaces allowed cell alignment and arrangement, which was not observed for the flat surface. Furthermore, cells in the flat surface were randomly distributed with limited cell to cell contact, whereas the convex and concave surfaces presented high degree of cell to cell contact.

#### 3.3.1 Cell circularity

**FIGURE 6:**
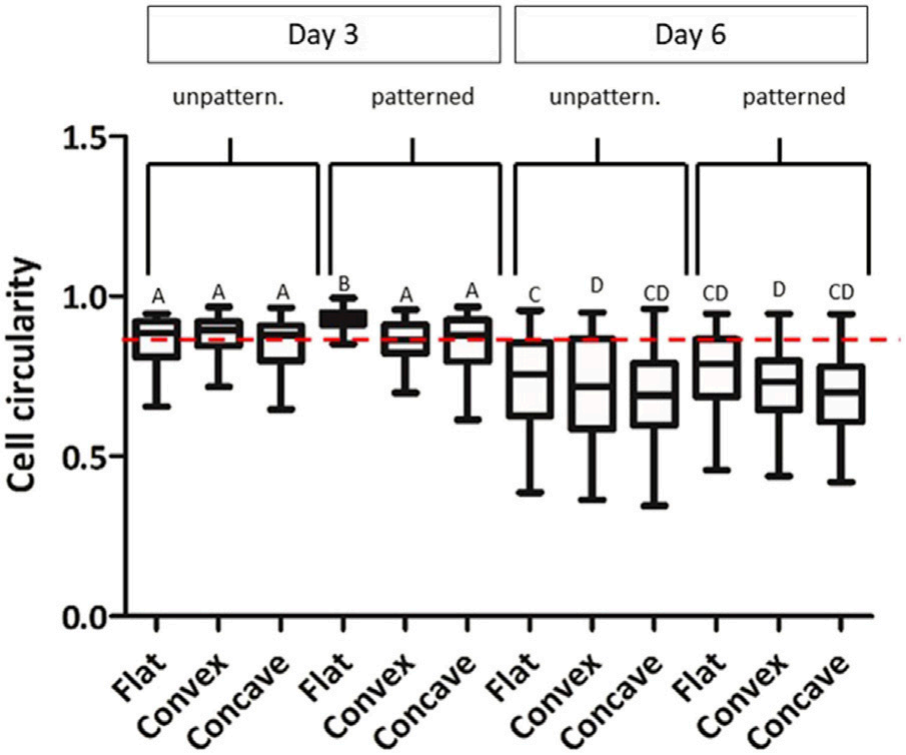
CEC cultured on collagen hydrogels for 3 and 6 days. Morphology analysis by cell circularity values of CEC culture on unpatterned and patterned hydrogels in concave, flat and convex hydrogels. * Different letter denotes significant differences (*p* < 0.05). Same letter denotes non-significant differences. **Red dotted line indicates a circularity value of 0.87.

We then analyzed the cell morphology of the cells shown in Figures 4, 5, analyzing cell circularity at days 3 and 6 (Figure 6). CEC exhibited a hexagonal and pentagonal morphology. A circle represents a value of 1 while a hexagon and pentagon have a circularity of 0.91 and 0.87 respectively. Our results showed that at day 3, cells presented a significantly higher circularity index and more homogeneous morphology (0.870 ± 0.071) than cells at day 6 (0.719 ± 0.132). At days 6, cells tended to decrease its circularity, although their polygonal shape was increased. Furthermore, cells cultured on patterned hydrogels presented higher circularity values than unpatterned hydrogels at day 3 and 6 of culture. However, there were only significant differences between patterned and unpatterned conditions in flat molds at day 3.

#### 3.3.2 Gene expression

**FIGURE 7:**
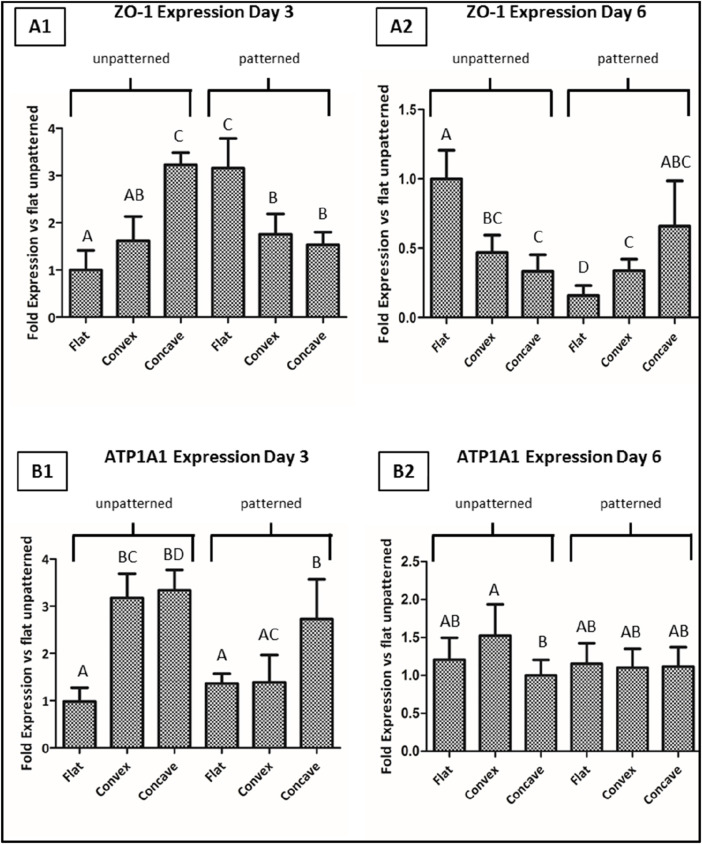
CEC cultured on collagen hydrogels for 3 and 6 days. Fold gene expression of ZO-1 at day 3 **(A1)** and 6 **(A2)**, and of ATP1A1 at day 3 **(B1)** and 6 **(B2)**, analyzed by quantitative real time-polymerase chain reaction (qPCR). * Different letter denotes significant differences. Same letter denotes non-significant differences.

Aiming to further characterize the effect of topography and curvature on CEC culture, the main cell tight junction (ZO-1) and the main pump function (ATP1A1) were analyzed by its gene expression, which is shown in Figure 7.

Regarding the ZO-1 gene expression, when cells were cultured on the unpatterned hydrogels, at 3 days of culture, CEC presented significantly higher levels of expression on any of the two curved hydrogels compared to the flat surface, having the highest degree of expression on the concave surfaces. For the same time point, the trend followed an opposite trend for the patterned structures, showing higher levels of expression for the flat surfaces. At day 6, in general the overall expression of the gene decreased, showing that the unpatterned surfaces presented highest levels of expression in the flat surfaces, whereas in the patterned surfaces, the expression was highest for the concave hydrogels.

Regarding the ATP1A1 expression for the unpatterned hydrogels at day 3, values showed significantly higher levels in the curved hydrogels compared to the flat hydrogels. Interestingly, for the patterned hydrogels, only concave hydrogels had significantly higher ATP1A1 expression compared to the convex and flat hydrogels. Lastly, at day 6 of culture, CEC cultured on the different hydrogels presented similar values in all cases, having the convex unpatterned hydrogels with the highest expression of ATP1A1, while the other conditions presented similar gene expression to the control condition.

### 3.4 Discussion

The aim of this work was to study the effect of the topography and curvature on an *in vitro* culture of corneal endothelial cells (CEC). It is well known that *in vivo* cell behavior depends on their surrounding microenvironment. Therefore, previous research has studied the advantages of culturing different type of cells on surfaces that mimic the native environment (Hu et al., 2014; Yim et al., 2010). More specifically, some studies have evaluated the effect of different patterned topographies in CEC culture, which ended in higher proliferation rate, maintenance of its characteristic cell morphology and improved cell function (Koo et al., 2014; Muhammad et al., 2015a; Teo et al., 2012). Ali et al. (2016) determined that Descemet’s membrane, which is in contact with *in vivo* CEC, consists of fibers with elevations and pores which form a hexagonal collagen network (Abrams et al., 2000). Therefore, previous groups evaluated patterns that mimicked native Descemet’s membrane structure like channels, wells, pillars and concentric circles (Koo et al., 2014; Muhammad et al., 2015a; Teo et al., 2012). However, the generation of these patterned molds requires the use of lithography-based techniques, which present some disadvantages, such as specialized equipment as well as the use of a difficult and expensive technique (Qin et al., 2010). Consequently, we selected the concentric circled micropattern and incorporated curvature to this pattern, which can be easily and rapidly generated using a conventional 3D-printer. Moreover, this pattern has also been used in a very recent assay for the formation of corneal patient-specific molds (Isaacson et al., 2018).

To begin, fibroblasts were cultured for 24 h on curved patterned hydrogels with low, medium and high LH hydrogels. Results showed that cells cultured on the 300 and 200 μm LH did not present any type of alignment, while this was only present in the 50 μm LH hydrogels. These results may indicate that medium and high LH hydrogels were too separated for cells to notice it. It is obvious that the cell behavior on substrates will greatly depend on the cell type that is being used. In this sense, neuronal cells, which may extend up to 100 μm, generally are able to align on different sized channels, whereas smaller cells such as chondrocytes, have more limited ability to extend along channel-like structures (Joergensen et al., 2015; Hoffman-Kim et al., 2010). In the case of fibroblasts, these cells generally have a size around few tens of microns, and hence their optimal interaction is the 50 μm sized LH. When the size of the LH was higher, cells were not able to sense the channel and behaved in a similar way to the smooth surfaces. Therefore, when LH is lower than cell size, cells became elongated and were distributed along the circle. Nevertheless, when LH was higher than cell size, cells morphology or distribution was not affected. This is in line with a previous report that seeded hepatocytes on different substrates, which concluded that spacing distances between patterns of 50 µm enhanced growth kinetics, compared to higher spacing which did not have an effect on cell growth (Lunova et al., 2016). Hepatocytes are an interesting cell source to compare with, as in their native morphology these also present a rather circular shaped morphology similar to our CEC and hence these results are in good agreement with our results. In contrast, the topography of low LH hydrogels determined the directionality of cell adhesion, which was also reported by a previous group (Teo et al., 2012).

CEC were then cultured on unpatterned and patterned, using the lowest LH, hydrogels and culturing cells at a native cell density (Muhammad et al., 2015a). Our results showed that the combination of topography and curvature affected CEC behavior.

As shown in Figures 4, 5, cells presented a more homogeneous morphology at short time (3 days), increasing its size at day 6. However, cells at day 6 presented a more polygonal morphology although cells were more elongated. To further characterize cell morphology and due to its characteristic polygonal shape, cell circularity was used for its evaluation (Figure 6). This parameter has been previously studied for the evaluation of *in vitro* CEC (Koo et al., 2014; Muhammad et al., 2015a; Beaulieu Leclerc et al., 2018). Surprisingly, the cell circularity decreased with time, which probably indicates that cells tended to have a rather elongated morphology in some cases. This was more evident in the cases in which cell confluence was reached, which mainly occurred in the flat surfaces. It was previously reported that the increase in cell passage reduced the CEC characteristic morphology, hence showing that prolonged culture periods *in vitro* may reduce their typical morphology (Palchesko et al., 2015).

In order to understand more detail the effect of topography and curvature, we analyzed two main markers, mainly ZO-1 and ATP1A1. ZO-1 is mainly related with tight junctions and hence its expression is based on how these junctions are established. It was previously shown that the tight junctions in CEC have an interesting behavior, showing that despite the overall cell morphology possess a rather hexagonal shape, the filopodia tend to have high surface area in order to have highest cell contact with other cells (He et al., 2016; Bade et al., 2018a). As shown in Figure 7, at day 3, we saw that the unpatterned surfaces showed higher expression for the concave and convex surfaces compared to the flat surfaces, presenting significant differences. This could be related to the fact that when cells are presented in a curved surface, cells have to adapt to different morphologies and small stresses are created on their surfaces, probably forcing them to have higher cell to cell contact between cells and hence increase the tight junction levels (Bade et al., 2018a). Nevertheless, when the topography is present, cells start arranging in a different manner. Cells no longer have the macro architecture of curvature, but rather have pits in the size range of their own cells, forcing cells to come in close contact among them. For this reason, we expected that at this point, the topography overruled the architecture and showed highest levels of expression on the flat surfaces. The fact that, in this case, the flat surfaces presented higher values of ZO-1 is probably related with the fact that cells have higher chances of falling in the different pits in a more homogeneous manner that in the curved substrates, where not all pits are identical and hence their behavior can be different. It was previously seen that a topography that forces cells to increase the tight junction connections increased as well ZO-1 levels (Koo et al., 2014; Muhammad et al., 2015a). Nevertheless, after 6 days, the behavior significantly changed, showing that in the unpatterned substrate ZO-1 levels were increased for the flat surfaces. This could be related with the fact that higher cell density is observed in this case and, hence, the chances of having higher ZO-1 levels were increased. Furthermore, it seemed that the curved surfaces decreased with time their effect. When analyzing the patterned substrates, we saw that, in general, the different substrates reduced the expression of ZO-1, especially for the flat surfaces. This could be related to the increased confluence levels showed for this condition, which forced cells to align on the pits, and despite the cell to cell contact existed, the fact that cells became elongated decreased the tight junctions between them. This could be hypothesized to be related with the Endothelial to Mesenchymal transition (EndMT), in which cells tend to reduce their endothelial behavior and go into a more fibroblastic behavior (Roy et al., 2015). Despite the levels of ZO-1 were also decreased for the concave and convex substrates, since these conditions did not present cells in confluence, cells did not completely adopt the fibroblastic character.

We then analyzed the expression of the pump function ATP1A1, which is related with their functionality and their capacity to exchange K^+^ and Na^+^ from the anterior chamber to the stroma. In this case, we observed that at day 3, for the unpatterned substrates, both the convex and the concave presented higher levels of ATP1A1 compared to the flat surface. This is in accordance with the fact that cells in their native environment are placed in a convex morphology and, hence, presenting this architecture enhances their functionality. This is very similar to the results obtained for ZO-1. It was previously seen that round surfaces were promising architectures for the culture of CEC (Yoshida et al., 2017; Kimoto et al., 2014). Similar results were observed for the patterned substrates at day 3, showing that both the concave and the flat surfaces followed the same trend, but was not observed for the convex substrates. It seems that for the ATP1A1 expression, unlike in the case of ZO-1, the curvature of the substrates overrules the topography of the substrates. Previous results showed accordingly that ZO-1 levels were deeply influenced by the topography, whereas the ATP1A1 levels, despite they were also affected by topography, its effect was lower (Koo et al., 2014). At day 6, the unpatterned substrates showed highest levels of expression for the convex substrates, which mimics the architecture of native cells and, hence, demonstrates that mimicking the native macro architecture had a beneficial effect on cell behavior. The flat and the concave surfaces behaved in a similar way among them, which could be related with the lower mimicking ability of their architectures and hence decreased their functionality. Finally, at day 6, the patterned substrates showed the same behavior in all three surfaces. It seems that as was previously shown, the culture of these cells may reduce their phenotypic activity with time (Roy et al., 2015). Hence, overall, it seems that these types of cultures are necessary for corneal endothelium tissue engineering; however, the culture time points are optimum at short times in order to prevent their loss of phenotypic activity *in vitro*. As it was mentioned earlier, this is related with the EndMT, which lead to rather fibroblastic phenotypes and hence a reduction in their functionality (Roy et al., 2015).

While the current work has established an interesting system to simultaneously expose CEC to environments similar to those found in native tissue, having both a micro-topography as well as the effect of curvature, cell are as well influenced by other factors. In this sense, the local microenvironment at the nano to micron level have not been considered in the current work. The understanding is that replicating such nano to micro topographies is complex and is not successfully achieved with the current methodology nor was the intention. Furthermore, the exact topography actually replicated is difficult to understand due to the complexity of visualizing the topographies in hydrogels. These nano to micro topographies modulates vital steps for cell behavior, allowing filopodia to explore the environment and allow cells to change phenotypic characters (Ventre et al., 2014; Ghibaudo et al., 2009). This is a relevant aspect to further combine in further work to allow integrating the sub-micron topography into the study and elicit positive response in the cell behavior. This may only be understood using the principles of mechanobiology and mechanotransduction, which is hindered in the current work only to the micro-groove architecture and the macro curvature of the structure. Several attempts have been done in order to understand the effect of nano-topographies on the CEC behavior (Muhammad et al., 2015b), but these are still far from native tissue and hence biomimetic patterns are required to mimic the vast complexity of native tissues (Xiong et al., 2019), which was beyond the scope of this work.

Nevertheless, despite of the need to understand the sub-micron topography, it has been established that the three-dimensional shape of the environment in scales larger than the cell size may also guide cell behavior (Kollmannsberger et al., 2011), which was indeed the purpose of the current work. In this sense, both the grooved structure and the curved morphology have seen to affect the temporal and spatial distribution of cells. The main rationale is that the underlying mechanism that is affected both by the micron sized groove and the curvature is related to the modulation of the actin cytoskeleton, although this still needs further work as was previously described (Baptista et al., 2019; Gouveia et al., 2017; Lee and Yang, 2017). Taking this into consideration, the discussion of whether the small grooved pattern has a dominant effect over the curvature of the whole hydrogel or the other way round, can only be explained by using the basics of physics and understanding single cell behavior. In this sense, the forces sensed in the micro-grooves exceed in general the forces observed in the macro curvature, forcing cells to overcome the environmental limitations and adopting rather elongated cells with eventually active filopodia. In this sense, it has previously hypothesized that micro patterns in the range of the ones proposed in the present work could affect directly cell behavior through a phenomenon known as curvotaxis (Pieuchot et al., 2018). Nevertheless, on the unpatterned structures, the curvature allowed cells to more freely adopt themselves and activate gap junctions, showing higher expression of the ZO-1 marker in the unpatterned hydrogels at short time points. It was previously hypothesized that concave substrates tend to induce higher expression of markers related to tight junctions (Soscia et al., 2013). Hence, the current state of the art is not able to depict the mechanism underlying such effects nor the dominant curvature in terms of cell regulation. As can be depicted, there is a general understanding and acceptance of the effect of curved substrates on cell behavior. While this is relevant and has biological effects, the inevitable curvature gradient in these substrates often limit the ability to obtain clear and direct mechanism of action. For this purpose, understanding single cell behavior on fiber like structures in order to avoid curvature gradients seems a plausible approach in a near future in order to understand the exact mechanism of action (Baptista et al., 2019).

Besides the curved structure of the eye, the human body accounts for a significant number of tissues with curved structures that mainly poses functionality owing the aforementioned curvature. The ability to fabricate such structures opens a new paradigm for tissue regeneration in which it has been show that curvature has a significant effect on cell behavior, although the exact mechanism remains unknown. Computational models have as well predicted their effects on cell morphology and their need in tissue engineering in near future, accounting the main mechanism of action on cell behavior for the actomyosin contractility (Callens et al., 2020; Bade et al., 2018b).

To summarize, our results demonstrate that cross-linked collagen hydrogels can be used as a platform for CEC culture. Moreover, topography and curvature culture seem to enhance CEC cell morphology and function at short time of culture with similar sizes to that of native cell. Further experimentation to understand the underlying mechanism as well as to observe longer time points and their effects not only at the gene expression level, but also in the protein expression level will be required to fully understand the possible clinical applications.

## 4 Conclusion

This work presents an innovative method to improve the culture of HCEC by combining for the first time a curved surface with microtopography, approaching CEC to sense a topographical environment similar to its native environment. For this purpose, a conventional 3D printer was used, which is an economic, rapid an easy technique, onto which collagen was replicated. The results showed that curved patterned substrates enhanced cell function and morphology at short time of periods of culture, as determined by the expression of characteristic markers ZO-1 and ATP1A1.

## Data Availability

The raw data supporting the conclusions of this article will be made available by the authors, without undue reservation.
